# Association of single nucleotide polymorphism rs3792876 in *SLC22A4* gene with autoimmune thyroid disease in a Chinese Han population

**DOI:** 10.1186/s12881-015-0222-x

**Published:** 2015-09-02

**Authors:** Xin Hou, Jinyuan Mao, Yushu Li, Jia Li, Weiwei Wang, Chenling Fan, Hong Wang, Hongmei Zhang, Zhongyan Shan, Weiping Teng

**Affiliations:** Department of Endocrinology and Metabolism, The First Hospital of China Medical University, Key Laboratory of Endocrine Diseases, Liaoning Province 110001 Shenyang, China; Key Laboratory of Endocrine Diseases, Liaoning Province Shenyang, China; Department of Geriatric Endocrinology and Metabolism, The First Hospital of China medical University, Shenyang, China

## Abstract

**Background:**

The autoimmune thyroid diseases (AITD), including Graves’ disease (GD) and Hashimoto’s thyroiditis (HT), are caused by interactions between susceptibility genes and environmental triggers. Single nucleotide polymorphisms (SNPs) of Solute carrier family 22, member 4 (*SLC22A4*) have been shown to be associated with several autoimmune diseases, including Crohn’s disease (CD) and rheumatoid arthritis (RA). The aim of this study is to investigate whether SNP rs3792876 in the *SLC22A4* gene is associated with GD, HT and AITD in a Chinese Han population.

**Methods:**

In this study, we collected specimens from 553 Chinese Han individuals of 92 AITD pedigrees in 10 cities in Liaoning province, China (80 GD pedigrees, 478 members; 12 HT pedigrees, 75 members). SNP rs3792876 was genotyped using the TaqMan allelic discrimination assay. Hardy-Weinberg Equilibrium tests were performed among founders of the pedigrees using Haploview software. Family-based association tests performed using FBAT software.

**Results:**

No deviation from Hardy-Weinberg equilibrium was observed (*p* > 0.05). There were not significant association between the *SLC22A4* gene polymorphism (rs3792876) and GD, HT and AITD was found.

**Conclusions:**

These results suggest a lack of association between the *SLC22A4* gene polymorphism rs3792876 and susceptibility to GD, HT and AITD in a Chinese Han population.

## Background

The autoimmune thyroid diseases (AITDs) are common organ-specific autoimmune diseases with an estimated prevalence of 1.5 % in general population [1]. They are characterized by infiltration of the thyroid with thyroid reactive lymphocytes and lead to two clinically opposing manifestations: Hashimoto’s thyroiditis (HT) with hypothyroidism and Graves’ disease (GD) affected by hyperthyroidism [2]. As most autoimmune disorders, AITD are complex conditions caused by interactions between genetic and environmental factors. Early family studies indicated that up to 50 % of the siblings of patients with AITD were thyroid antibody positive in contrast to ∼ 15 % in the general population [3], suggesting a strong genetic impact on the etiology of AITD. Since human leukocyte antigen (*HLA*) loci were first associated with GD [4], more than thirty chromosome regions [5] and a number of immune-related genes [6, 7] have been implicated in the genetic susceptibility to AITDs.

Among these genetic factors, the chromosome 5q31 region is of special interest because both our previous GD family study [8] and a Japanese AITD affected sib-pair study recognized 5q31 as an AITD susceptibility locus [9]. In addition, genetic variants in the 5q31 region have been shown to be responsible for autoimmune related disorders, such as Crohn’s disease (CD) [3], atopic dermatitis (AD) [4] and bronchial asthma [5], by both classic genetic linkage and powerful genome-wide association (GWAS) studies. This chromosome region contains a cluster of cytokine and immune-related genes including *IL4*, *IL13* and *IRF1*, suggesting a significant involvement of 5q31 in human cytokine and autoimmune regulation [10, 11]. However, limited studies have been carried out to further investigate the relationship between candidate genes in 5q31 and AITDs, which makes it still unclear of the underlying mechanism for 5q31 genetic susceptibility to AITDs.

The *SLC22A4* gene is located on chromosome 5q31 and encodes the organic cation transporter OCTN1. It is highly expressed in immune-related organs and tissues. *SLC22A4* mRNA expression is induced by proinflammatory stimuli [12]. Genetic polymorphisms have been found to affect *SLC22A4* gene expression and to be associated with autoimmune diseases such as rheumatoid arthritis (RA),CD and type 1 diabetes (T1D) [12–14]. All these findings suggest the essential role of *SLC22A4* in the autoimmune regulatory pathway. It maybe related to autoimmune diseases in the development of autoimmunity in secondary lymphoid organs [12]. Studies of an intron 1 single nucleotide polymorphism (SNP) rs3792876 in *SLC22A4* showed that this polymorphism affected binding of a transcription regulator, runt-related transcription factor 1 (RUNX-1) [15, 16] and might regulate the transcription of *SLC22A4* [12]. Several other recent publications have revealed that polymorphisms affecting RUNX-1 binding sites may be associated with a variety of autoimmune diseases such as psoriasis [15] and systemic lupus erythematosus (SLE) [16]. Despite all these evidences of involvement of *SLC22A4* in human autoimmune disease, the association of *SLC22A4* with AITD has never been studied. We hypothesized that *SLC22A4* might be involved in genetic susceptibility of AITD through the transportation of small organic molecules (such as immune regulatory factors), and such involvement might be mediated by a SNP located in a RUNX1-binding sequence in *SLC22A4* affecting the expression of *SLC22A4*. In this study we collected AITD pedigrees (GD pedigrees and HT pedigrees), genotyped rs3792876 and assess the genetic susceptibility of this *SLC22A4* polymorphism to GD, HT and AITD in our Chinese Han populations.

### Methods

#### Subjects ascertainment

In this study 92 Chinese Han AITD pedigrees (80 GD pedigrees and 12 HT pedigrees) with 553 individuals were recruited in Liaoning Province, China. Pedigrees were ascertained through GD or HT probands at the outpatient clinic of Department of Endocrine Diseases of the First Hospital of China Medical University. Family history was subsequently confirmed by at least one affected first-degree family member. HT was diagnosed as follows: 1) medical history or clinical biochemistry evidence of hypothyroidism requiring thyroid hormone replacement therapy, and 2) presence of autoantibodies to thyroidperoxidase, with or without antibodies to thyroglobulin. GD was diagnosed as follows: 1) medical history or clinical biochemistry evidence of hyperthyroidism, 2) diffused goiter, and 3) the presence of at least one of the following manifestations: positive thyroid stimulating hormone (TSH) receptor antibody tests, diffusely increased ^131^I (iodine-131) uptake in the thyroid gland or presence of exophthalmos. All other family members were classified as unaffected, whether their thyroid autoantibody tests were positive or negative. All diagnoses were made by at least two independent endocrine physicians. Research protocols were approved by the medical ethics committee of the China Medical University and conformed to the guidelines set forth by the Declaration of Helsinki. Written informed consents were acquired from all of the participants or their guardians. Peripheral venous blood samples were collected from all of the participants for DNA preparations and biochemical measurements.

### Genotyping

Genomic DNA was isolated from peripheral white blood cells using the DNA purification kit (QIAgen, Hilden, Germany) according to the manufacturer’s instructions. The rs3792876 SNP was genotyped by Taqman allelic discrimination assay on a Roche LC480 Real-Time PCR System (Roche Diagnostics, Penzberg, Germany). The SNP was genotyped using a predesigned TaqMan SNP Genotyping Assay (Applied Biosystems; assay ID C_3170428_10). Amplification was performed in a total of 10ul containing 30 ng of template DNA according to the manufacturer’s standard program protocol. 5 % of the samples were randomly chosen to confirm the genotypes by direct sequencing for quality control.

### Statistical methods

The Hardy-Weinberg Equilibrium was test among founders using Haploview4.2 [17]. Family-based association tests (FBAT) were performed using the FBAT software [18] (V2.0.3 Harvard School of Public Health, Departments of Biostatistics and Environmental Health, Program for Population Genetics, Boston, Massachusetts, USA). All of the tests were performed under the assumption of an additive model, with a null hypothesis of no linkage and no association. A *P* < 0.05 was considered statistically significant. Differences in genotype frequencies between patients and cases were calculated by chi-square or Fisher’s exact test when necessary.

## Result

Among 92 AITD pedigrees (553 individuals), 29 families (32 %) had2patients, 39 (42 %) had 3and17 (18 %) had 4, 5 (5 %) had 5 and 2 (2 %) had 6. Among the 80 GD pedigrees (478 individuals), 20 (25 %) had 1affected first-degree relatives, 37 (46 %) had 2, 16 (20 %) had 3, 5 (6 %) had 4 and 2 (3 %) had 5. In total, there were 201 GD patients (45males and156 females; male/female ratio of 1:3.47; mean age of 43.72 ± 14.31 years) and 277 first-degree relatives (136 males and141 females, mean age of 48.95 ± 16.98 years). Among the 12 HT pedigrees (75 individuals), 9(75 %) had 1affected first-degree relatives, 2(17 %) had 2, and 1(8 %) had 3. There were 29 HT patients (8 males and 21 females, male/female ratio of 1:2.62, mean age of 42.45 ± 19.85 years) and 46 first-degree relatives (18 males and 28 females, mean age of 47.55 ± 16.75 years). The patients’ general condition was list in Table 1.

**Table 1:** The clinical manifestation and biochemical characterization of GD, HT and AITD

	GD pedigree		HT pedigree		AITD pedigrees	
	GD patients	Controls	HT patients	Controls	AITD patients	Controls
Gender (M/F)	45/156 (1:3.47)	136/141 (1:1.04)	8/21 (1/2.62)	18/28 (1:1.56)	54/176 (1:3.25)	153/170 (1:1.11)
Age (year)	43.72 ± 14.31	48.95 ± 16.98	42.45 ± 19.85	47.55 ± 16.75	43.60 ± 15.17	48.40 ± 17.21
TSH (mIU/L)	0.10 (0.06-1.83)	1.26 (0.79-2.06)	2.04 (0.23-9.06)	2.45 (1.45-3.33)	0.23 (0.01-2.32)	1.37 (0.83-2.30)
FT_4_ (pmol/L)	25.81 ± 18.55	16.64 ± 3.07	18.79 ± 9.87	16.50 ± 2.01	24.91 ± 17.82	16.62 ± 2.94
FT_3_ (pmol/L)	7.58 ± 6.72	4.17 ± 0.89	4.71 ± 1.89	4.38 ± 0.97	7.21 ± 6.39	4.20 ± 0.90
TPOAb (IU/ml)	368.50 (46.85-1000)	10.10 (10.00-29.20)	1000.00 (458.75-1000.00)	10.60 (10.00-158.50)	503.50 (53.13-1000.00)	10.10 (10.00-33.35)
TgAb (IU/ml)	59.95 (20.00-460.00)	20.00 (20.00-94.70)	80.60 (31.18-3000.00)	20.00 (20.00-45.70)	59.95 (20.00-519.50)	20.00 (20.00-20.00)

No deviation from Hardy-Weinberg equilibrium was detected among all founders (*P* > 0.05). The genotyping call rate was 99.6 % and the sequencing confirmation rate was 100 %. In total, 80 GD, 12 HT and 92 AITD pedigrees were genotyped for the SNP rs3792876. The T allele frequency of rs3792876 was 21 % in GD pedigrees and 20 % in AITD pedigrees. In addition, we performed a FBAT; however, there was no significant association detected (*p* > 0.05) (Table 2). We also compared the genotype of rs3792876 in GD, HT and AITD patients and controls, there was no significant association (*p* > 0.05) (Table 3).

**Table 2:** FBAT analysis of the association between GD, HT and AITD and rs37928

Disease	Family	S-E(S)	Var(S)	Z	*P*
GD	39	1.40	34.91	0.24	0.813
HT	1	-	-	-	-
AITD	40	0.65	35.47	0.11	0.913

Family: Number of families that are actually informative for the test. S: test statistics for the observed number of transmitted alleles. E(S): expected value of S under the null hypothesis (i.e., no linkage or association)

**Table 3:** Comparison of genotype of rs3792876 in GD, HT and AITD patients and controls

	GD pedigree		HT pedigree		AITD pedigree	
	GD patients	Controls	HT patients	Controls	AITD patients	Controls
CC	123	180	23	35	146	215
CT	70	85	6	9	76	94
TT	8	12	0	2	8	14
P	0.633	0.532	0.571			

## Discussion

Our previous study mapped a susceptible locus of GD in chromosomal 5q31 regions [8]. A maximum LOD score over 4 indicated a strong linkage between a GD phenotype and polymorphic genetic markers in this region. We focused on this region in a subsequent study because this region contains many immune related genes associated with autoimmune disorders or traits such as CD, asthma, arthritis and T1D. We tested the linkage between SNPs in 5q31 genes *IL4*, *IL13*, *IRF1* and *UGRP1* in Chinese GD patients [19]. However, a negative association was observed.

In this study, we chose *SLC22A4* as candidate gene based on current knowledge on its molecular function and genetic predisposition studies. *SLC22A4* encodes an organic cation transporter mainly expressed in the liver, kidney, intestine and other organs. It is critical for the elimination of many endogenous small organic cations as well as a wide array of drugs and environmental toxins. A functional intronic polymorphism rs3792876 has been studied in several different ethnic groups for an association with immune disorders including CD, T1D and RA. However, inconsistent results have been obtained. The association of rs3792876 with RA was originally reported in a Japanese population. The researchers suggested an allelic difference of rs3792876 in affinity to RUNX1, a transcriptional regulator in the hematopoietic system [12]. This association was replicated in other Japanese populations [20, 21] but not in Caucasian and Spanish groups [22, 23]. The SNP rs3792876 was also shown to genetically predispose patients to T1D [13] and CD [24]. However, these findings were never confirmed.

Before the current study, the association between rs3792876 and GD, HT and AITD has never been assessed. Our negative results may reflect the real condition that this variant may confer to limit or even no contribution to etiology of GD, HT, and AITD. Although previous studies suggested rs3792876’s involvement into immune disorders, due to pathophysiological difference between AITD and other autoimmune diseases, it’s highly possible that *SLC22A4* may be genetically totally unrelated to GD, HT, or AITD.

Potential limitations of this study must also be addressed. First, the sample size of our study is relatively small, which may not have the efficient statistical power to detect a weak genetic effect, resulting in a fluctuated estimation. Second, we evaluated association of only one common polymorphism of *SLC22A4* gene with susceptibility to GD, HT and AITD, there might be still other unidentified SNPs which could influence the development of GD, HT or AITD. Third, our pedigrees are all Han Chinese. Genetic background is a very important issue in genetic association study and may affect both association strength and direction. Rs3792876 allelle frequencies differ greatly among different ethnic groups with highest MAF in East Asian (>0.2) and much lower in other populations (<0.1). Therefore, more studies with larger sample size, multiple ethnic groups and more candidate genes are needed to further study the molecular genetic basis underlying chromosome region 5p31 in predisposition to GD, HT and AITD.

## Conclusion

In this study we assessed the association of *SLC22A4* gene polymorphism rs3792876 with Chinese Han GD, HT and AITD patients, our results suggested lack of association between rs3792876 and GD, HT and AITD in the Chinese Han population.
